# Editorial: Early detection and management of neurocognitive disorders

**DOI:** 10.3389/fpsyt.2023.1199593

**Published:** 2023-04-24

**Authors:** Ram J. Bishnoi

**Affiliations:** ^1^Department of Psychiatry and Behavioral Neurosciences, University of South Florida, Tampa, FL, United States; ^2^USF Health Byrd Alzheimer's Institute, University of South Florida, Tampa, FL, United States

**Keywords:** neurocognitive deficit, cognition, early - biomarkers, aging, disability

This Special Topic of Aging Psychiatry section of Frontiers in Psychiatry presents work illustrating the importance and various methods of early identification of cognitive impairment. The prompt and timely detection of cognitive impairment is not only allows for precise diagnosis and adequate treatment but also ensure shared decision-making concerning healthcare and other life plans. A wide array of methods to identify and characterize early cognitive changes are showcased through this section.

The article by Jin et al. overview current evidence for a neurobehavioral syndrome, Mild Behavioral Impairment (MBI), as a risk indicator in preclinical Alzheimer's disease (AD). MBI is a relatively newer concept and believed to precede cognitive decline. The authors examined psychometric, neuroimaging, and neuropathologic evidence to highlight utility of MBI in early identification of AD. Marrero-Polegre et al. attempted to identify subtle cognitive changes by measuring visual processing speed, a basic visual attention function that influences global cognition. In a group of cognitively healthy community dwelling elderly individuals, lower visual processing speed correlated with subjective cognitive decline. Jang et al. investigated whether a Virtual reality (VR)-based cognitive assessment program can be as effective as traditional methods to screen individuals for cognitive impairment. This is a small step in the direction of wider use of VR based technology in detection and treatment of neurocognitive disorders. In this experiment, VR based assessment outperformed existing screening tools. Lombardi et al. examined a public health aspect of early identification of neurocognitive disorder i.e., by providing education to primary care providers. The educational intervention not only improved the early detection but also the management practices. This multicentric study conducted throughout Italy provide evidence and a template for implementation of such programs in other countries. Subjective cognitive and memory decline can precede cognitive decline. Pacifico et al. explored the associations of subjective cognitive decline and subjective memory decline with objective cognitive and memory performances, disability, and depressive symptoms. Their observation of disability mediating the associations of objective performance and subjective report highlight the need for further inquiry in this direction. Lastly, in an assessment of neuroimaging-based methodology to improve identification and quantification of small vessel disease, Gaubert et al. compared available tools in multicenter context. Their results provide recommendations for future studies about segmentation tools for white matter hyperintensities.

The special topic research articles offer a comprehensive range of methods and techniques to enhance the early identification of cognitive impairment, which can potentially contribute to the advancement of neurocognitive disorder care. Moreover, these research articles may either reinforce ongoing research or stimulate novel research avenues.

## Author contributions

The author confirms being the sole contributor of this work and has approved it for publication.

